# Higher serum uric acid levels are associated with an increased risk of carotid atherosclerosis in 67,256 adults

**DOI:** 10.3389/fnut.2026.1752079

**Published:** 2026-02-11

**Authors:** Renhao Chen, Yifan Geng, Mingyi Lin, Lei Qiu, Yi Ning, Qionggui Zhou

**Affiliations:** 1 Key Laboratory of Tropical Translational Medicine of Ministry of Education, School of Public Health, Hainan Medical University, Haikou, Hainan, China; 2 The Key Lab of Tropical Cardiovascular Diseases Research of Hainan Province, Haikou, China; 3 Hainan General Hospital (Hainan Affiliated Hospital of Hainan Medical University), Haikou, China

**Keywords:** carotid atherosclerosis, cross-sectional study, dose–response relationship, serum uric acid, sex-specific association

## Abstract

**Background:**

The association between serum uric acid (SUA) and carotid atherosclerosis (CAS) remains controversial, particularly regarding its independence from traditional risk factors. This cross-sectional study aimed to investigate this relationship and its dose–response pattern in 67,256 adults.

**Methods:**

We conducted a cross-sectional analysis of 67,256 participants who underwent health check-up at Beijing MJ Healthcare Center between 2012 and 2022. Data collected included demographics, physical exams, laboratory tests, and carotid ultrasound. Participants were divided into quartiles based on baseline SUA levels. Multivariate logistic regression analysis was performed with four models to assess the association between SUA and CAS, as well as its subtypes, including increased carotid intima-media thickness (cIMT) and carotid plaque (CP). Additionally, SUA was treated as a continuous variable, and a Restricted Cubic Spline (RCS) model was fitted to explore the dose–response relationship. Subgroup and sensitivity analyses were conducted to examine the heterogeneity and robustness of our findings.

**Results:**

The prevalence of CAS was 28.8%. Each standard deviation (per-SD) increase in SUA was significantly associated with CAS risk (OR = 1.22, 95% CI: 1.20–1.24, Model 1), (OR = 1.07, 95%CI: 1.04–1.10, Model 4). When SUA was analyzed by quartiles, the Q4 group showed a higher CAS risk (OR = 1.85, 95%CI: 1.76–1.95, Model 1), (OR = 1.19, 95%CI: 1.10–1.28, Model 4). Consistently, across different CAS subtypes, higher levels of SUA were associated with an increased risk of both increased cIMT and CP. The RCS model revealed a linear dose–response relationship in the total population, with CAS risk increasing when SUA exceeded 324 μmol/L. Subgroup analysis demonstrated sex-specific associations: higher SUA (Q4) was significantly linked to CAS in females (OR = 1.34, 95%CI: 1.14–1.58) but not in males (OR = 1.04, 95%CI: 0.92–1.17). There is a linear dose–response relationship for females and a nonlinear trend for males. Both age groups (<60 and ≥60 years) showed increased CAS risk with elevated SUA. Sensitivity analyses excluding individuals with hypertension, diabetes, or dyslipidemia yielded consistent results (per-SD: OR = 1.08, 95%CI: 1.04–1.12; Q4 group: OR = 1.19, 95%CI: 1.06–1.33).

**Conclusion:**

The prevalence of CAS was high in the Beijing health check-up population, and high levels of SUA were significantly associated with an increased risk of CAS, particularly in females.

## 1 Introduction

Cardiovascular diseases (CVDs) remain a leading global health burden, with projections indicating substantial increases in prevalence and mortality by 2050 (1). In China, atherosclerotic CVD accounts for over 40% of deaths (2). As a window to systemic atherosclerosis, the occurrence and development of carotid atherosclerosis (CAS) are closely related to a variety of metabolic factors. Current research primarily focuses on various subtypes of CAS, including increased carotid intima-media thickness (cIMT), carotid plaque (CP), and carotid stenosis (CS). In 2020, the global prevalence of increased cIMT in people aged 30–79 years was estimated to be 27.6%, CP was estimated to be 21.1%, and CS was estimated to be 1.5% (3). In the Chinese population aged 20 years and older, the weighted prevalence of all subtypes was 26.2% for increased cIMT, 21% for CP, 0.56% for CS, and 0.15% for moderate to severe CS (4). However, the various subtypes of CAS exhibit a continuum in pathological progression, and their occurrence and development may be influenced by partially shared etiological factors and risk factors. Among these, the role of serum uric acid (SUA) has garnered significant attention in recent years.

SUA is the end product of purine nucleotide metabolism, and its abnormal levels are closely associated with various CVDs (5–8). Studies indicate that SUA contributes to the atherosclerotic process by promoting oxidative stress, inflammatory responses, and endothelial dysfunction (5, 9, 10). Notably, even when SUA levels are at the upper limit of the normal range (309–357 μmol/L), elevated levels remain associated with the onset and progression of CVDs (11).

Current research on the association between SUA and different subtypes of CAS yields inconsistent conclusions. Some studies suggested an association between elevated SUA and increased cIMT (12, 13), and a cross-sectional study conducted in multiple provinces in Chinese indicated that elevated SUA levels were associated with an increased risk of developing all three subtypes of CAS (4). However, a multi-rural community-based cohort study in South Korea found no association between increased cIMT and SUA levels in both sexes (14). Overall, existing evidence primarily focuses on middle-aged and elderly populations, lacking systematic evaluation across broader adult age groups (≥18 years). Furthermore, most relevant studies have been conducted in Western, Japanese, and Korean populations, with limited high-quality data available for the Chinese population.

Therefore, we aimed to investigate the association between SUA and CAS and its subtypes, and to test a dose–response relationship in adults based on a large health check-up population in Beijing. Additionally, we performed a series of subgroup and sensitivity analyses to test the heterogeneity and robustness of our results.

## 2 Materials and methods

### 2.1 Study design and participants

We used a cross-sectional study design and included study subjects who participated in medical check-up and completed serum uric acid tests and carotid ultrasound examinations at the Beijing MJ Healthcare Center from 2012 to 2022. For those who participated in health check-up several times during the 11 years, only the latest health check-up of the same participants were included to ensure that the data analyzed better reflect the current health status of the population. In total, 69,594 participants were eligible for this study. After excluding those aged less than 18 years (*N* = 20), those with unclear diagnosis of CAS (*N* = 1755), and those with outliers of covariates such as height, BMI, and blood pressure exceeding Mean±3SD (*N* = 563), this study finally included 67,256 participants for analysis.

We collected information about the study population through questionnaires, including general demographic information (age, sex, height, weight, etc.) and lifestyle (smoking status, alcohol drinking, etc.). All physical examinations, laboratory tests, and ancillary investigations were performed by qualified healthcare professionals using certified medical equipment in strict accordance with standardized operating procedures. CAS was examined by carotid ultrasound using a Philips EPIQ7 color Doppler ultrasound system (equipped with a C5-1 convex array probe, scanning frequency of 3.5 MHz). The diagnostic criteria for CAS subtypes are as follows: cIMT of 1.0 mm or greater is defined as thickening (15). CP is defined as meeting any of the following morphological criteria: a focal structure within a specific arterial segment exhibiting intima-media thickness ≥1.5 mm, or thickening ≥0.5 mm or exceeding 50% compared to the surrounding vessel wall (16). The severity of CS is graded based on the percentage reduction in lumen diameter: less than 50% is mild stenosis, 50 to 69% is moderate stenosis, and 70% or greater is classified as severe stenosis (17). For the primary analysis, a composite binary endpoint of CAS was defined as the presence of any of the above conditions (increased cIMT, CP, or CS of any degree).

### 2.2 Statistical analysis

Categorical variables were described using numbers (%), and comparisons between groups were made using the Chi-square test or Fisher’s exact test. Continuous variables were described using mean ± standard deviation (Mean ± SD), and comparisons between groups were made using the independent *T*-test or analysis of variance.

SUA levels were standardized using the per-standard deviation (per-SD) method. Specifically, each SUA measurement was centered by subtracting the population mean and then scaled by dividing by the standard deviation. Participants were categorized according to the quartiles of the baseline SUA level. The subjects were divided into Q1 (*n* = 16,646), Q2 (*n* = 16,875), Q3 (*n* = 16,846), and Q4 (*n* = 16,889). Logistic regression models were used to estimate odds ratios (ORs) and 95% confidence intervals (CIs) for risk of CAS and its subtypes (increased cIMT and CP) across different SUA categories with four models: Model 1: unadjusted; Model 2: adjusted for age and sex; Model 3: adjusted for age, sex, smoking status, and alcohol drinking; Model 4: adjusted for age, sex, smoking status, alcohol drinking, body mass index (BMI), systolic blood pressure (SBP), diastolic blood pressure (DBP), fasting blood glucose (FBG), triglyceride (TG), total cholesterol (TC), creatinine and estimated glomerular filtration rate (eGFR).

To assess the dose–response relationship between SUA and CAS, a logistic regression model with restricted cubic splines (RCS) was fitted, treating SUA as a continuous variable. Subgroup analyses were stratified by sex and age (<60 years vs. ≥ 60 years) to examine the heterogeneity of the associations between SUA and CAS. To clarify the role of SUA in different pathological stages of CAS, we categorized CAS into two independent binary outcome variables: increased cIMT and CP, then analyzed their respective associations with SUA. To assess the robustness of the findings and rule out potential reverse causality, we conducted a sensitivity analysis based on first health check-up data from the total population. Meanwhile, Previous studies had identified hypertension, diabetes, and dyslipidemia as risk factors for CAS (3, 4, 13). To assess the robustness of the results, we conducted sensitivity analyses, excluding: (1) participants with hypertension (SBP ≥ 140 mmHg and/or DBP ≥ 90 mmHg, or a documented history of hypertension with ongoing antihypertensive medication use) (18); (2) participants with diabetes (FBG ≥ 7 mmol/L or a documented history of diabetes with ongoing hypoglycemic medication use) (19); (3) participants with dyslipidemia (TG ≥ 2.3 mmol/L, or TC ≥ 6.2 mmol/L or HDL-c < 1.0 mmol/L or LDL-c ≥ 4.1 mmol/L, or ongoing use of lipid-lowering medications) (20); and (4) participants with hypertension, diabetes, or dyslipidemia.

All statistical analyses were performed using R (version 4.2.2). Two-sided *p* < 0.05 was considered statistically significant.

## 3 Result

**Figure 1:**
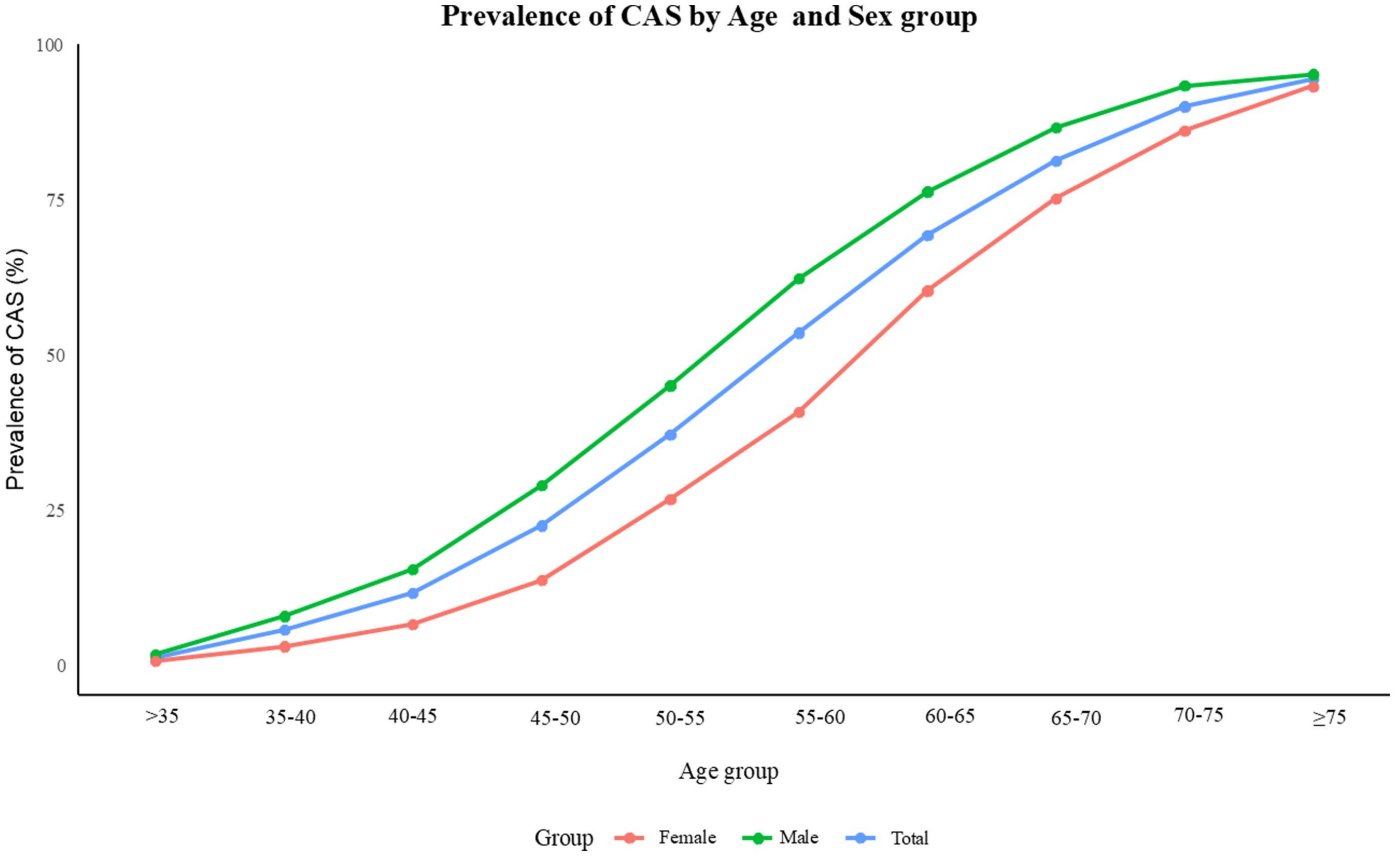
Prevalence trend of carotid atherosclerosis by age and sex groups among study participants (males in green, females in red).

A total of 67,256 participants were included, and the overall prevalence of CAS was 28.8% (19,394/67,256). The mean age of participants was 47 ± 12 years. The baseline characteristics of the study population stratified by CAS status are presented in Table 1. Significant differences were observed in all variables between the two groups (all *p* < 0.001). Individuals with CAS tended to be older and were more likely to be males. They also had higher rates of current smoking and drinking compared to those without CAS. In addition, those with CAS generally exhibited higher levels of BMI, SBP, DBP, creatinine, SUA, FBG, TG, TC, and LDL-c, while having lower HDL-c levels and eGFR. The overall prevalence of CAS increased with age and was higher in males than in females (Figure 1).

**Table 1:** Baseline characteristics of study participants.

Characteristic	Overall (*N* = 67,256)	No-CAS (*N* = 47,862)	CAS (*N* = 19,394)	*p*-value
Sex (%)				<0.001
Male	37,473 (55.7%)	24,666 (51.5%)	12,807 (66.0%)	
Female	29,783 (44.3%)	23,196 (48.5%)	6,587 (34.0%)	
Age (year)	47 ± 12	43 ± 9	58 ± 11	<0.001
Height (cm)	168 ± 8	168 ± 8	167 ± 8	<0.001
Weight (kg)	69 ± 13	68 ± 13	71 ± 12	<0.001
BMI (kg/m^2^)	24.4 ± 3.4	24.1 ± 3.4	25.2 ± 3.1	<0.001
SBP (mmHg)	117 ± 16	114 ± 14	125 ± 17	<0.001
DBP (mmHg)	72 ± 11	71 ± 11	75 ± 11	<0.001
SUA (μmol/L)	331 ± 88	326 ± 89	343 ± 84	<0.001
FBG (mmol/L)	5.72 ± 1.20	5.54 ± 0.94	6.19 ± 1.59	<0.001
TG (mmol/L)	1.46 ± 1.09	1.38 ± 1.07	1.63 ± 1.11	<0.001
TC (mmol/L)	4.74 ± 0.92	4.69 ± 0.88	4.86 ± 1.00	<0.001
HDL-c (mmol/L)	1.38 ± 0.41	1.41 ± 0.42	1.32 ± 0.36	<0.001
LDL-c (mmol/L)	3.09 ± 0.84	3.04 ± 0.80	3.22 ± 0.92	<0.001
creatinine (μmol/L)	72.0 ± 17.0	70.8 ± 15.8	74.9 ± 19.3	<0.001
eGFR(mL/min/1.73m^2^)	99.6 ± 21.1	101.9 ± 21.5	93.9 ± 18.9	<0.001
Smoking status (%)				<0.001
Never	48,271 (71.8%)	35,894 (75.0%)	12,377 (63.8%)	
Past	3,280 (4.9%)	1,656 (3.5%)	1,624 (8.4%)	
Current	15,705 (23.4%)	10,312 (21.5%)	5,393 (27.8%)	
Alcohol Drinking (%)				<0.001
Never	51,989 (77.3%)	38,066 (79.5%)	13,923 (71.8%)	
Past	1,395 (2.1%)	767 (1.6%)	628 (3.2%)	
Current	13,872 (20.6%)	9,029 (18.9%)	4,843 (25.0%)	

Data are shown as Mean ± SD or number (%); CAS, carotid atherosclerosis; BMI, body mass index; SBP, systolic blood pressure; DBP, diastolic blood pressure; SUA, serum uric acid; FBG, fasting blood glucose; TG, triglycerides; TC, total cholesterol; HDL-c, high-density lipoprotein cholesterol; LDL-c, low-density lipoprotein cholesterol; eGFR, estimated glomerular filtration rate.

**Figure 2:**
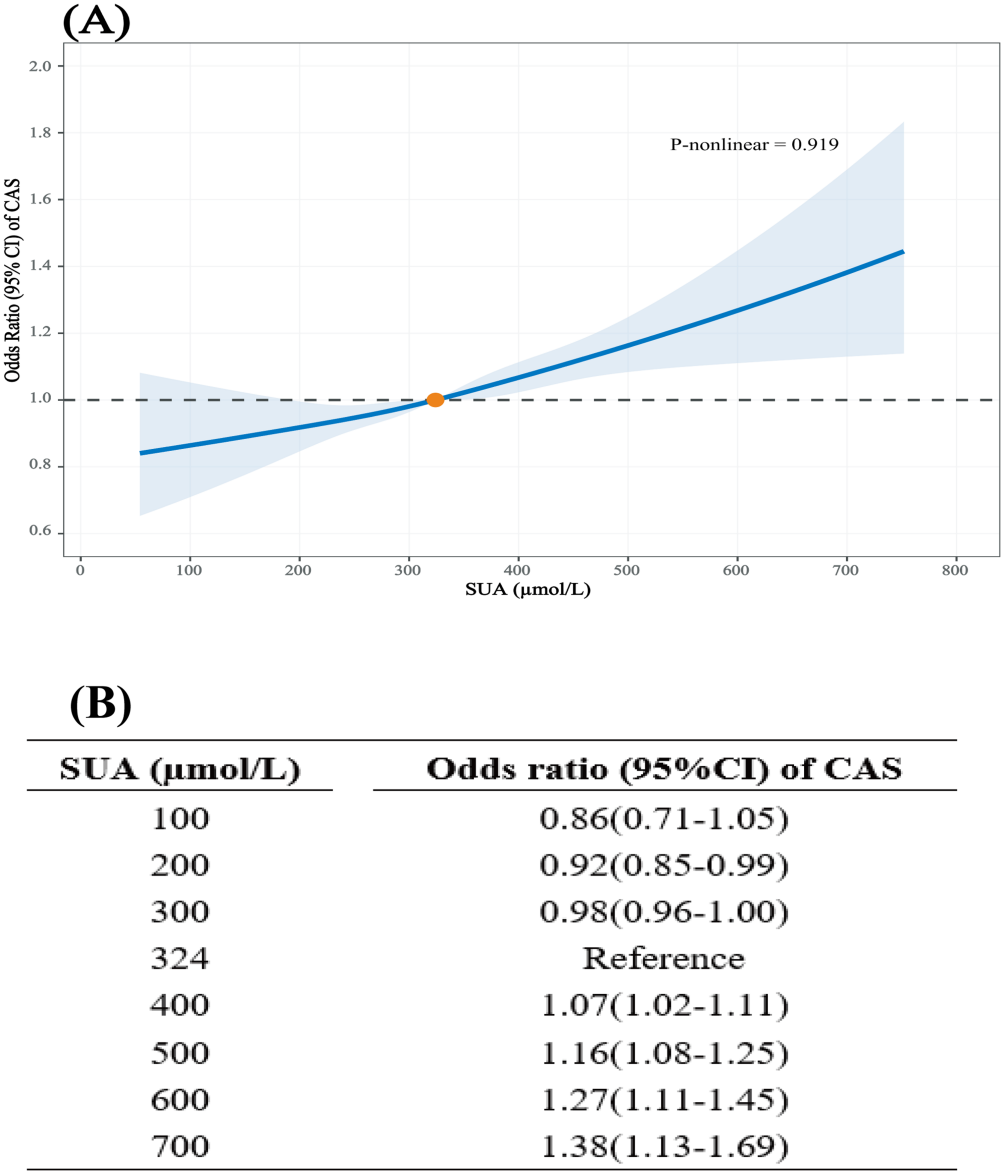
Dose–response association between serum uric acid and carotid atherosclerosis. Adjusted for sex, age, smoking status, alcohol drinking, BMI, SBP, DBP, FBG, TG, TC, creatinine, and eGFR. **(A)** RCS curve showing the adjusted OR for CAS across SUA levels. **(B)** Selected point estimates.

This study found a significant association between SUA levels and CAS risk in all models. The risk of CAS increased with per-SD change in SUA: Model 1 (OR = 1.22, 95%CI: 1.20–1.24), Model 2 (OR = 1.12, 95%CI: 1.09–1.15), Model 3 (OR = 1.12, 95%CI: 1.09–1.15) and Model 4 (OR = 1.07, 95%CI: 1.04–1.10). Compared to the reference group (Q1), SUA levels in Q2, Q3, and Q4 were associated with an increased risk of CAS when unadjusted with ORs of 1.56 (95%CI: 1.49–1.64), 1.82 (95%CI: 1.74–1.92), and 1.86 (95%CI: 1.76–1.95), respectively. After adjusting for age, sex, smoking status, alcohol drinking, BMI, SBP, DBP, FBG, TG TC, creatinine and eGFR in Model 4, SUA levels in Q4 were associated with a higher CAS risk (OR = 1.19, 95%CI: 1.10–1.28) (Table 2). Similar associations were observed across different CAS subtypes, with higher SUA levels being significantly associated with increased risks of CP (Model4 OR: 1.21, 95% CI: 1.10–1.32) and increased cIMT (Model4 OR: 1.18, 95% CI: 1.08–1.30) (Supplementary Table S1). The RCS model showed a linear dose–response relationship between SUA and CAS after adjusting for all covariates (*P* for nonlinearity = 0.919). When SUA levels exceeded 324 μmol/L, the risk of CAS increased with increasing SUA levels (Figure 2A). Compared to those with an SUA level of 324 μmol/L, the adjusted OR (95% CI) was 1.16 (1.08–1.25) and 1.38 (1.11–1.69) for SUA levels of 500 μmol/L and 700 μmol/L, respectively (Figure 2B).

**Table 2:** Logistic regression analyses of associations between serum uric acid and carotid atherosclerosis.

SUA Category	Model 1		Model 2		Model 3		Model 4	
	OR(95% CI)	*P*-value	OR(95% CI)	*P*-value	OR(95% CI)	*P*-value	OR(95% CI)	*P*-value
SUA (per-SD)	1.22 (1.20–1.24)	<0.001	1.12 (1.09–1.15)	<0.001	1.12 (1.09–1.15)	<0.001	1.07 (1.04–1.10)	<0.001
SUA								
Q1 [27,265]	Reference		Reference		Reference		Reference	
Q2 [265,324]	1.56 (1.49–1.64)	<0.001	1.09 (1.02–1.16)	0.010	1.09 (1.02–1.16)	0.011	1.04 (0.97–1.11)	0.300
Q3 [324,388]	1.82 (1.74–1.92)	<0.001	1.10 (1.03–1.18)	0.005	1.11 (1.03–1.19)	0.004	1.04 (0.96–1.11)	0.300
Q4 [388,800]	1.85 (1.76–1.95)	<0.001	1.33 (1.24–1.43)	<0.001	1.33 (1.23–1.43)	<0.001	1.19 (1.10–1.28)	<0.001
*P* for trend		<0.001		<0.001		<0.001		<0.001

OR, odds ratio; CI, confidence interval.

Model 1: no covariates were adjusted.

Model 2: adjusted for sex and age.

Model 3: adjusted for sex, age, smoking status, and alcohol drinking.

Model 4: adjusted for sex, age, smoking status, alcohol drinking, BMI, SBP, DBP, FBG, TG, TC, creatinine and eGFR.

**Figure 3:**
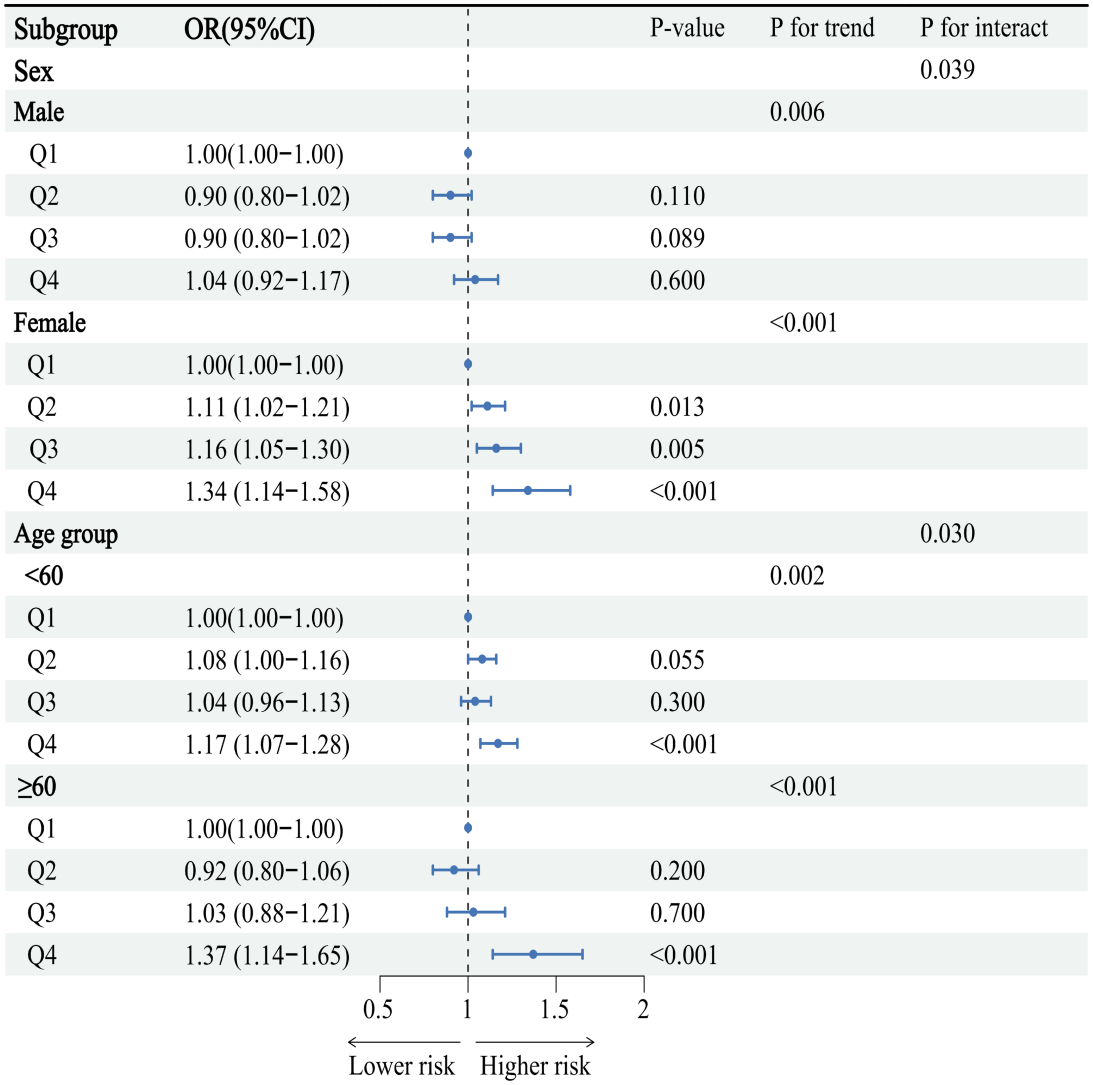
Subgroup analysis of the association between serum uric acid and carotid atherosclerosis by sex and age. Adjusted for sex, age, smoking status, alcohol drinking, BMI, SBP, DBP, FBG, TG, TC, creatinine, and eGFR.

**Figure 4:**
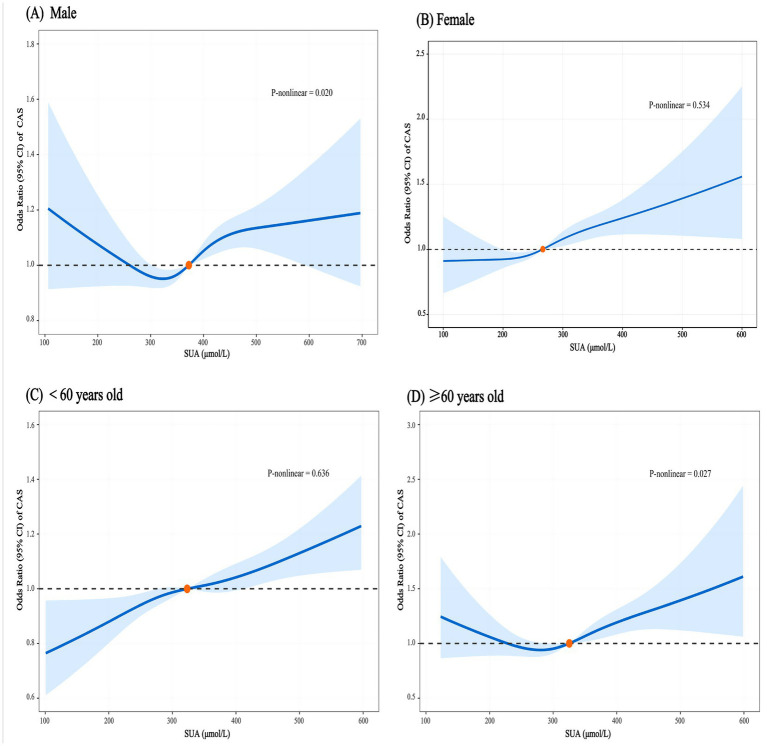
Subgroup analysis of the dose–response association between serum uric acid and carotid atherosclerosis. **(A)** Male. **(B)** Female. **(C)** Age <60 years. **(D)** Age ≥60 years. Adjusted for sex, age, smoking status, alcohol drinking, BMI, SBP, DBP, FBG, TG, TC, creatinine and eGFR.

Subgroup analyses showed a sex-specific pattern between SUA and CAS (*P* for interaction = 0.039), with high levels of SUA (Q4) increasing the risk of CAS in females (OR = 1.34, 95%CI: 1.14–1.58) but not males (OR = 1.04, 95%CI: 0.92–1.17). In different age groups, high levels of SUA (Q4) significantly increased the risk of CAS in both <60 years (OR = 1.17, 95%CI: 1.07–1.28) and ≥60 years (OR = 1.37, 95%CI: 1.14–1.65) (*P* for interaction = 0.030) (Figure 3). Meanwhile, the RCS model showed that after adjusting for all covariates, there was a significant linear dose–response relationship between SUA and CAS in females (*P* for nonlinearity = 0.534). When the SUA level exceeded 268 μmol/L, the risk of CAS increased with increasing SUA level (Figure 4B). However, in males, there was a nonlinear dose–response relationship between SUA and CAS (*P* for nonlinearity = 0.020), and the association between SUA and CAS existed only at SUA levels between 372 and 590 μmol/L, while the association was not statistically significant when SUA was at higher or lower levels (Figure 4A). High levels of SUA significantly increased the risk of CAS in both <60 years and ≥60 years. When SUA levels exceeded 412 μmol/L in those <60 years and 325 μmol/L in those ≥60 years, the risk of CAS increased with increasing SUA in both age groups (Figures 4C,D).

Sensitivity analysis results based on first health check-up data indicated that for each standard deviation increase in SUA, the risk of CAS increased by 4% (Model 4: OR: 1.04, 95% CI: 1.01–1.07). Compared with the lowest SUA level, the highest level was associated with a 9% increased risk of CAS (Model 4: OR: 1.09, 95% CI: 1.00–1.18) (Supplementary Table S2). RCS results indicated a dose–response relationship between SUA and CAS (*P* for nonlinearity = 0.121), the risk of CAS increases when SUA levels exceed 516 μmol/L (Supplementary Figure S1A). Similarly, sensitivity analysis, which excluded patients with hypertension, diabetes, dyslipidemia, and any of the three diseases, showed results consistent with the main findings (Supplementary Table S2; Supplementary Figure S1).

## 4 Discussion

In this large cross-sectional analysis, we found that higher levels of SUA were significantly and positively associated with the risk of CAS and its subtypes in the Beijing health check-up population. Subgroup analyses revealed a sex-specific difference in the association between SUA levels and CAS, with high levels of SUA significantly increasing the risk of CAS in females, but not in males. Various sensitivity analyses showed that the association between SUA and CAS remained robust.

The prevalence of CAS and the association between SUA levels and CAS and its subtypes (CP and increased cIMT) in our study are consistent with previous studies. Jingzhu Fu et al. enrolled 10,733,975 participants across 31 provinces in China. The findings indicated that the prevalence of CAS was higher in older adults and males, and elevated SUA levels were a risk factor for CP and increased cIMT (4). We observed a sex-specific difference in the association between SUA and CAS, with higher levels of SUA increasing the risk of CAS in females but not in males. Previous studies reported similar sex differences. For example, one study found an association between UA and CAS, which was more significant in females than in males (21); another also reported that the effect of SUA on atherosclerosis and CAS was higher in females than in males (22). In addition, Li L et al. found that SUA level predicted the development of CAS independently of traditional risk factors only in females, but not in males (23). The sex differences may arise from estrogen in females promotes renal excretion of SUA and maintains low levels of SUA in the body (22). Therefore, females are more sensitive to elevated SUA levels than males. High SUA levels can stimulate oxidative stress and promote the development of CAS. Our study found that after excluding patients with hypertension, diabetes, and dyslipidemia, the association between SUA levels and CAS remained significant, which is consistent with previous studies. A study found that higher SUA levels were associated with increased cIMT in non-hypertensive patients (5). Furthermore, Shuzo Takayama et al. found that there was a significant correlation between UA levels and increased cIMT in the Japanese population aged 60 years and older, and this association was also significant in males and females without metabolic syndrome (12).

The mechanisms underlying the association between SUA and CAS have not been fully elucidated. However, several hypotheses and studies had suggested that high SUA levels may contribute to the onset and development of CAS through multiple pathways: (1) Oxidative stress: oxidative stress is a key factor in the development of CAS (24, 25). Studies had indicated that SUA can promote oxidative activity and induce intracellular oxidative stress. Oxidative stress promotes CAS by inducing endothelial cell dysfunction, inflammation in inflammatory cells (e.g., macrophages), platelet aggregation, and low-density lipoprotein oxidation (26). (2) Endothelial dysfunction: hyperuricemia disrupts the asymmetric dimethylarginine (ADMA)/dimethylarginine dimethylaminohydrolase-2 (DDAH-2) axis in endothelial cells (ECs), leading to endothelial cell dysfunction and accelerating atherosclerosis (27). (3) Lipid metabolic disorders: SUA levels are significantly associated with dyslipidemia (28–30). Patients with hyperuricemia are often accompanied by hypercholesterolemia, hypertriglyceridemia, and low HDL cholesterol, and these dyslipidemias promote CAS (29). These multiple mechanisms interact and accelerate CAS progression.

This study’s strength lies in its use of the Beijing MJ Healthcare Center Database, which is a large-scale health check-up database with the advantages of a large sample size (67,256). We not only used SUA as a categorical variable to investigate the association between different SUA groups and CAS, but also included SUA as a continuous variable in the RCS model to further investigate the dose–response relationship between SUA and CAS. In addition, subgroup and sensitivity analyses were conducted to evaluate the heterogeneity and robustness of our findings. However, our study has several limitations. First, as a cross-sectional study, our ability to infer causality is limited. This raises the possibility of reverse causation or unmeasured confounding factors influencing the observed associations. To mitigate this, we employed robust statistical methods, including multivariate logistic regression with comprehensive adjustment for confounders (e.g., age, sex, lifestyle factors, and cardiometabolic variables) and RCS models to explore dose–response relationships. Second, SUA levels in this study were based on is a single measurement, which may be inaccurate due to influences from short-term factors, such as diet, dehydration, medication, or physical activity, and future studies should use mean values from multiple measurements. Thirdly, because Beijing has a high level of economic development and health literacy, and the study population consisted of individuals actively undergoing health check-up, caution should be exercised when generalizing the results to other populations. To address this, we conducted sensitivity analyses and detailed subgroup analyses. These findings generate hypotheses for future longitudinal cohort studies to confirm the temporal relationship and explore underlying mechanisms, thereby building on the strengths of this cross-sectional design.

## 5 Conclusion

This study found that the overall prevalence of CAS was high among Chinese adults, and a high level of SUA was significantly associated with the risk of CAS, especially in females. Our findings suggest that SUA levels should be actively monitored, and that timely intervention in high-risk groups may reduce the risk of CAS.

## Data Availability

The data were derived from the database of Beijing MJ Health Screening Center and could not be shared publicly due to patient privacy and confidentiality agreements. Data access is available upon reasonable request by contacting the corresponding author, subject to ethical approval. Requests to access these datasets should be directed to ningyi@muhn.edu.cn; qiongguiz@muhn.edu.cn.
