# Supplementary material for: Higher serum uric acid levels are associated with an increased risk of carotid atherosclerosis in 67,256 adults
Source: Front Nutr. 2026 Feb 11;13:1752079. doi: 10.3389/fnut.2026.1752079 (PMC12932208; doi:10.3389/fnut.2026.1752079)
Supplement: Supplementary file 1 [file Table_1.docx]

Table S1. Logistic regression analyses of associations between serum uric acid and different carotid atherosclerosis subtypes.

| **SUA Category** | **Model 1** | | **Model 2** | | **Model 3** | | **Model 4** | |
| --- | --- | --- | --- | --- | --- | --- | --- | --- |
|  | **OR(95% CI)** | ***P*-value** | **OR(95% CI)** | ***P*-value** | **OR(95% CI)** | ***P*-value** | **OR(95% CI)** | ***P*-value** |
| **SUA AND CP** |  |  |  |  |  |  |  |  |
| **SUA (per-SD)** | 1.21 (1.19-1.23) | <0.001 | 1.13 (1.09-1.16) | <0.001 | 1.12 (1.09-1.15) | <0.001 | 1.08 (1.05-1.12) | <0.001 |
| **SUA** |  |  |  |  |  |  |  |  |
| Q1 [27,264) | Reference |  | Reference |  | Reference |  | Reference |  |
| Q2 [264,322) | 1.55 (1.47-1.64) | <0.001 | 1.04 (0.96-1.13) | 0.300 | 1.04 (0.96-1.13) | 0.300 | 1.01 (0.93-1.09) | 0.900 |
| Q3 [322,387) | 1.82 (1.72-1.93) | <0.001 | 1.08 (0.99-1.17) | 0.075 | 1.08 (1.00-1.17) | 0.056 | 1.04 (0.95-1.13) | 0.400 |
| Q4 [387,800] | 1.83 (1.73-1.94) | <0.001 | 1.32 (1.21-1.44) | <0.001 | 1.31 (1.20-1.43) | <0.001 | 1.21 (1.10-1.32) | <0.001 |
| *P* for trend |  | <0.001 |  | <0.001 |  | <0.001 |  | <0.001 |
| **SUA AND increased cIMT** | | | | | | | | |
| **SUA (per-SD)** | 1.24 (1.22-1.27) | <0.001 | 1.11 (1.08-1.15) | <0.001 | 1.11 (1.08-1.15) | <0.001 | 1.07 (1.04-1.11) | <0.001 |
| **SUA** |  |  |  |  |  |  |  |  |
| Q1 [27,263) | Reference |  | Reference |  | Reference |  | Reference |  |
| Q2 [263,322) | 1.64 (1.54-1.75) | <0.001 | 1.09 (1.01-1.18) | 0.035 | 1.09 (1.00-1.18) | 0.038 | 1.04 (0.96-1.13) | 0.300 |
| Q3 [322,386) | 1.99 (1.87-2.13) | <0.001 | 1.10 (1.01-1.20) | 0.025 | 1.11 (1.02-1.21) | 0.021 | 1.05 (0.96-1.14) | 0.300 |
| Q4 [386,800] | 2.05 (1.92-2.18) | <0.001 | 1.30 (1.19-1.42) | <0.001 | 1.30 (1.19-1.42) | <0.001 | 1.18 (1.08-1.30) | <0.001 |
| *P* for trend |  | <0.001 |  | <0.001 |  | <0.001 |  | <0.001 |
| OR=Odds Ratio, CI=Confidence Interval, CP=carotid plaque, cIMT=carotid intima–media thickness; | | | | | | | |  |
| Model 1: no covariates were adjusted; | | | | | | | |  |
| Model 2: adjusted for sex and age; | | | | | | | |  |
| Model 3: adjusted for sex, age, smoking status, and alcohol drinking; | | | | | | | |  |
| Model 4: adjusted for sex, age, smoking status, alcohol drinking, BMI, SBP, DBP, FBG, TG, TC, creatinine and eGFR. | | | | | | | |  |

Table S2. Sensitivity analyses of serum uric acid and carotid atherosclerosis.

| **SUA Category** | **Model 1** | | **Model 2** | | **Model 3** | | **Model 4** | |
| --- | --- | --- | --- | --- | --- | --- | --- | --- |
|  | **OR(95% CI)** | ***P*-value** | **OR(95% CI)** | ***P*-value** | **OR(95% CI)** | ***P*-value** | **OR(95% CI)** | ***P*-value** |
| **Based on first health check-up data** | | | | | | | | |
| \| **SUA (per-SD)** \| 1.19 (1.17-1.21) \| <0.001 \| 1.09 (1.06-1.12) \| <0.001 \| 1.09 (1.06-1.12) \| <0.001 \| 1.04 (1.01-1.07) \| 0.009 \| \| --- \| --- \| --- \| --- \| --- \| --- \| --- \| --- \| --- \| \| **SUA** \|  \|  \|  \|  \|  \|  \|  \|  \| \| Q1 [27,263) \| Reference \|  \| Reference \|  \| Reference \|  \| Reference \|  \| \| Q2 [263,322) \| 1.57 (1.49-1.66) \| <0.001 \| 1.10 (1.03-1.18) \| 0.006 \| 1.10 (1.03-1.18) \| 0.006 \| 1.05 (0.98-1.13) \| 0.200 \| \| Q3 [321,386) \| 1.80 (1.70-1.89) \| <0.001 \| 1.09 (1.02-1.18) \| 0.017 \| 1.10 (1.02-1.18) \| 0.015 \| 1.02 (0.95-1.11) \| 0.500 \| \| Q4 [386,860] \| 1.75 (1.66-1.85) \| <0.001 \| 1.24 (1.14-1.33) \| <0.001 \| 1.23 (1.14-1.33) \| <0.001 \| 1.09 (1.00-1.18) \| 0.045 \| \| *P* for trend \|  \| <0.001 \|  \| <0.001 \|  \| <0.001 \|  \| 0.087 \| | | | | | | | | |
| **Excluding participants with hypertension** | | | | | | | | |
| **SUA (per-SD)** | 1.23 (1.20-1.25) | <0.001 | 1.12 (1.08-1.15) | <0.001 | 1.11 (1.08-1.14) | <0.001 | 1.08 (1.05-1.11) | <0.001 |
| **SUA** |  |  |  |  |  |  |  |  |
| Q1 [27,263) | Reference |  | Reference |  | Reference |  | Reference |  |
| Q2 [263,321) | 1.52 (1.43-1.60) | <0.001 | 1.06 (0.99-1.14) | 0.110 | 1.06 (0.99-1.14) | 0.120 | 1.02 (0.95-1.10) | 0.600 |
| Q3 [321,384) | 1.84 (1.74-1.94) | <0.001 | 1.07 (0.99-1.16) | 0.079 | 1.07 (0.99-1.16) | 0.069 | 1.01 (0.93-1.10) | 0.800 |
| Q4 [384,800] | 1.88 (1.78-1.99) | <0.001 | 1.27 (1.17-1.38) | <0.001 | 1.27 (1.17-1.37) | <0.001 | 1.16 (1.06-1.27) | <0.001 |
| *P* for trend |  | <0.001 |  | <0.001 |  | <0.001 |  | <0.001 |
| **Excluding participants with diabetes** | | | | | | | | |
| **SUA (per-SD)** | 1.23(1.21,1.25) | <0.001 | 1.14(1.11,1.17) | <0.001 | 1.14(1.11,1.17) | <0.001 | 1.07(1.04,1.10) | <0.001 |
| **SUA** |  |  |  |  |  |  |  |  |
| Q1 [27,265) | Reference |  | Reference |  | Reference |  | Reference |  |
| Q2 [265,324) | 1.53 (1.45-1.62) | <0.001 | 1.07 (1.00-1.15) | 0.047 | 1.07 (1.00-1.15) | 0.048 | 1.02 (0.95-1.09) | 0.600 |
| Q3 [324,388) | 1.85 (1.76-1.95) | <0.001 | 1.14 (1.06-1.23) | <0.001 | 1.14 (1.06-1.23) | <0.001 | 1.04 (0.96-1.12) | 0.300 |
| Q4 [388,800] | 1.89 (1.80-2.00) | <0.001 | 1.38 (1.28-1.50) | <0.001 | 1.37 (1.27-1.49) | <0.001 | 1.18 (1.09-1.29) | <0.001 |
| *P* for trend |  | <0.001 |  | <0.001 |  | <0.001 |  | <0.001 |
| **Excluding participants with dyslipidemia** | | | | | | | | |
| **SUA (per-SD)** | 1.29 (1.26-1.31) | <0.001 | 1.10 (1.07-1.14) | <0.001 | 1.10 (1.07-1.14) | <0.001 | 1.08 (1.04-1.12) | <0.001 |
| **SUA** |  |  |  |  |  |  |  |  |
| Q1[27,255) | Reference |  | Reference |  | Reference |  | Reference |  |
| Q2[255,307) | 1.52 (1.42-1.62) | <0.001 | 1.08 (1.00-1.18) | 0.063 | 1.08 (0.99-1.17) | 0.066 | 1.05 (0.97-1.15) | 0.200 |
| Q3[307,369) | 2.00 (1.88-2.13) | <0.001 | 1.14 (1.05-1.25) | 0.002 | 1.14 (1.05-1.25) | 0.002 | 1.11 (1.01-1.21) | 0.027 |
| Q4[369,800] | 2.14 (2.01-2.27) | <0.001 | 1.28 (1.17-1.41) | <0.001 | 1.28 (1.17-1.40) | <0.001 | 1.21 (1.10-1.34) | <0.001 |
| *P* for trend |  | <0.001 |  | <0.001 |  | <0.001 |  | <0.001 |
| **Excluding participants with hypertension, diabetes or dyslipidemia** | | | | | | | | |
| **SUA (per-SD)** | 1.28 (1.25-1.32) | <0.001 | 1.11 (1.07-1.15) | <0.001 | 1.10 (1.06-1.15) | <0.001 | 1.08 (1.04-1.12) | <0.001 |
| **SUA** |  |  |  |  |  |  |  |  |
| Q1 [27,250) | Reference |  | Reference |  | Reference |  | Reference |  |
| Q2 [250,303) | 1.51 (1.40-1.63) | <0.001 | 1.11 (1.01-1.22) | 0.024 | 1.11 (1.01-1.22) | 0.026 | 1.09 (0.99-1.20) | 0.084 |
| Q3 [303,365) | 2.03 (1.89-2.19) | <0.001 | 1.15 (1.04-1.27) | 0.006 | 1.15 (1.04-1.27) | 0.006 | 1.10 (0.99-1.22) | 0.073 |
| Q4 [365,800] | 2.16 (2.01-2.33) | <0.001 | 1.28 (1.15-1.42) | <0.001 | 1.27 (1.15-1.42) | <0.001 | 1.19 (1.06-1.33) | 0.002 |
| *P* for trend |  | <0.001 |  | <0.001 |  | <0.001 |  | 0.004 |
| OR=Odds Ratio; CI=Confidence Interval; | | | | | | | |  |
| Model 1: no covariates were adjusted; | | | | | | | |  |
| Model 2: adjusted for sex and age; | | | | | | | |  |
| Model 3: adjusted for sex, age, smoking, and alcohol drinking; | | | | | | | |  |
| Model 4: adjusted for sex, age, smoking status, alcohol drinking, BMI, SBP, DBP, FBG, TG, TC, creatinine and eGFR. | | | | | | | |  |


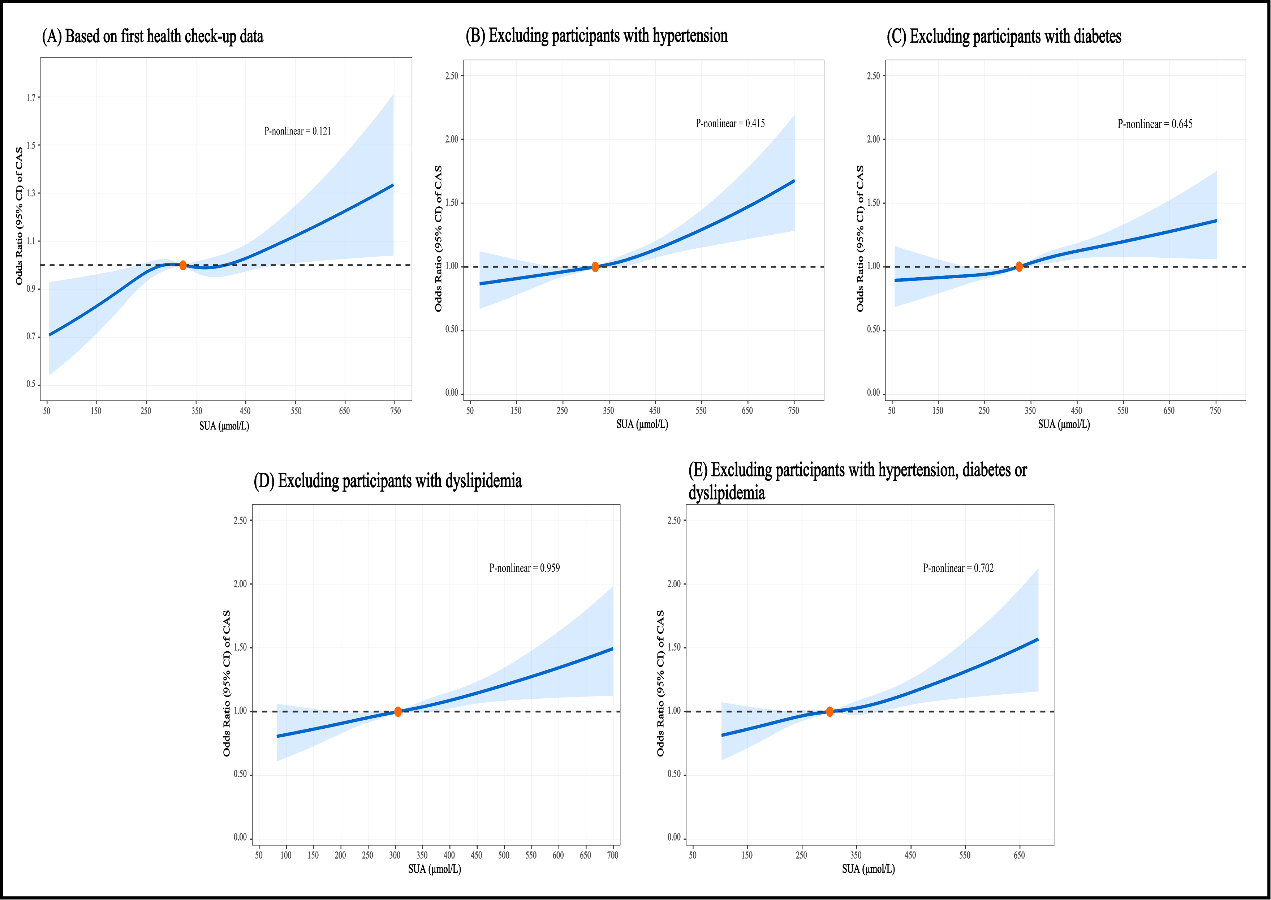


Figure S1. Sensitivity analysis of the dose-response association between serum uric acid and carotid atherosclerosis. (A) Based on first health check-up data. (B) Excluding participants with hypertension. (C) Excluding participants with diabetes. (D) Excluding participants with dyslipidemia. (E) Excluding participants with hypertension, diabetes or dyslipidemia.

Adjusted for sex, age, smoking status, alcohol drinking, BMI, SBP, DBP, FBG, TG, TC, creatinine and eGFR.
